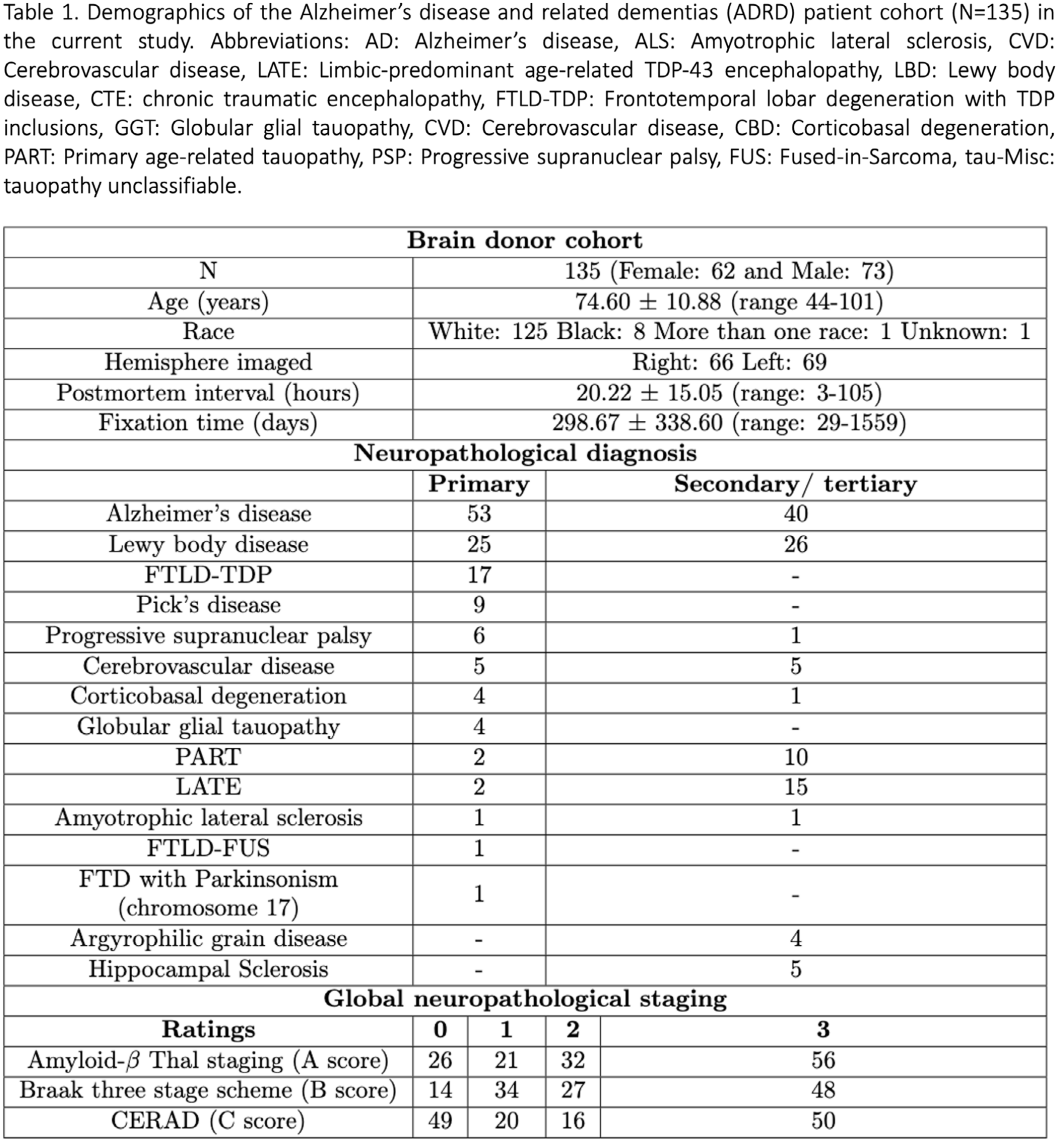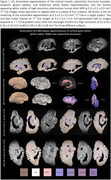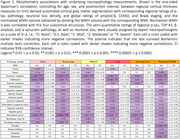# High‐resolution 7 tesla postmortem MRI for quantitative analysis of structure‐pathology correlations in neurodegenerative diseases

**DOI:** 10.1002/alz.091569

**Published:** 2025-01-09

**Authors:** Pulkit Khandelwal, Michael Tran Duong, Shokufeh Sadaghiani, Sydney A Lim, Amanda E Denning, Eunice Chung, Sadhana Ravikumar, Sadhana Ravikumar, Sanaz Arezoumandan, Claire Peterson, Madigan Bedard, Noah Capp, Ranjit Ittyerah, Elyse Migdal, Grace Choi, Emily Kopp, Bridget Loja Patino, Eusha Hasan, Jiacheng Li, Alejandra Bahena, Karthik Prabhakaran, Gabor Mizsei, Marianna Gabrielyan, Theresa Schuck, Winifred Trotman, John L. Robinson, Daniel T Ohm, Eddie B Lee, John Q Trojanowski, Corey T McMillan, Murray Grossman, David J Irwin, John A. Detre, Dylan M Tisdall, Sandhitsu R. Das, Laura E.M. Wisse, David A Wolk, Paul A. Yushkevich

**Affiliations:** ^1^ Penn Image Computing and Science Laboratory (PICSL), University of Pennsylvania, Philadelphia, PA USA; ^2^ University of Pennsylvania, Philadelphia, PA USA; ^3^ Department of Neurology, University of Pennsylvania, Philadelphia, PA USA; ^4^ Penn Frontotemporal Degeneration Center, Department of Neurology, Perelman School of Medicine, University of Pennsylvania, Philadelphia, PA USA; ^5^ Department of Neurology, Perelman School of Medicine, University of Pennsylvania, Philadelphia, PA USA; ^6^ Digital Neuropathology Laboratory, University of Pennsylvania, Philadelphia, PA USA; ^7^ Perelman School of Medicine at the University of Pennsylvania, Philadelphia, PA USA; ^8^ Frontotemporal Degeneration Center, Department of Neurology, Perelman School of Medicine, University of Pennsylvania, Philadelphia, PA USA; ^9^ Department of Pathology & Laboratory Medicine, University of Pennsylvania, Philadelphia, PA USA; ^10^ Institute on Aging, University of Pennsylvania, Philadelphia, PA USA; ^11^ Center for Neurodegenerative Disease Research, University of Pennsylvania, Philadelphia, PA USA; ^12^ Department of Pathology and Laboratory Medicine, Philadelphia, PA USA; ^13^ Center for Neurodegenerative Disease Research, Perelman School of Medicine, University of Pennsylvania, Philadelphia, PA USA; ^14^ Perelman School of Medicine, University of Pennsylvania, Philadelphia, PA USA; ^15^ Penn FTD Center, University of Pennsylvania, Philadelphia, PA USA; ^16^ Penn Alzheimer’s Disease Research Center, University of Pennsylvania, Philadelphia, PA USA; ^17^ Frontotemporal Degeneration Center, University of Pennsylvania, Philadelphia, PA USA; ^18^ Perelman School of Medicine, University og Pennsylvania, Philadelphia, PA USA; ^19^ Department of Clinical Sciences Lund, Lund University, Lund, Lund Sweden; ^20^ Lund University, Lund Sweden; ^21^ Penn Memory Center, University of Pennsylvania, Philadelphia, PA USA; ^22^ Department of Pathology and Laboratory Medicine, Alzheimer’s Disease Center, Perelman School of Medicine, University of Pennsylvania, Philadelphia, PA USA

## Abstract

**Background:**

Postmortem MRI allows brain anatomy to be examined at high‐resolution linking pathology with morphometric measurements. However, automated methods for analyzing postmortem MRI are not well developed. We present a deep learning‐based framework for automated segmentation of cortical mantle, subcortical structures (caudate, putamen, globus pallidus, and thalamus), white matter hyperintensities (WMH), and normal appearing white matter in (n=135) postmortem human brain tissue specimens (Table 1) imaged at 0.3 mm^3^ T2w 7T spanning Alzheimer’s disease and related dementias. We show generalizing capabilities across unseen images acquired at 0.28 mm^3^ and 0.16 mm^3^ T2*w 7T FLASH sequence. We report associations between localized cortical thickness and volumetric measurements across key regions and semi‐quantitative neuropathological ratings.

**Method:**

A deep learning model was trained on manually segmented images to produce automated whole‐brain hemisphere segmentations (Figure 1) with a post‐hoc topological correction step to delineate buried sulcus. We report regional patterns of association between localized cortical thickness at 16 anatomical locations and neuropathology ratings of regional measures of p‐tau, neuronal loss; global amyloid‐β, Braak staging, and CERAD ratings obtained from histology data in a subset (n=82) with AD continuum diagnoses. We correlate subcortical volumetry and regional cortical thickness with WMH burden (Figure 2) for the entire cohort (n=135). All analyses include age, sex, and postmortem interval as covariates.

**Result:**

Tau pathology in Braak regions play an important role in cortical atrophy and cognitive decline in AD. Significant negative correlations (Figure 2) between p‐tau and cortical thickness were found in angular gyrus and midfrontal regions. Cortical thickness showed significant negative correlation with neuronal loss in Brodmann area (BA) 35 and entorhinal cortex (ERC), and with Braak staging in midfrontal, ERC and BA35, regions consistent with high p‐tau uptake in PET imaging with cortical thickness on MRI. High WMH volume disrupts structural and functional connectivity impacting memory. Significant negative correlation of WMH volume with thickness in posterior cingulate and superior temporal regions was observed.

**Conclusion:**

Our automated postmortem MRI framework provides geometrically accurate segmentations of several key brain regions. Our analysis linking morphometry and pathology measurements demonstrated that automated segmentation and analysis of postmortem MRI can complement and inform antemortem neuroimaging studies.